# Use of n-3 PUFAs can decrease the mortality in patients with systemic inflammatory response syndrome: a systematic review and meta-analysis

**DOI:** 10.1186/s12944-015-0022-5

**Published:** 2015-03-31

**Authors:** Xiao Wan, Xuejin Gao, Jingcheng Bi, Feng Tian, Xinying Wang

**Affiliations:** Department of General Surgery, Jinling Hospital, School of Medicine, Nanjing University, Nanjing, Jiangsu Province China; Clinical College of South Medical University, Guangdong Province, China

**Keywords:** Polyunsaturated fatty acids, Sepsis, SIRS, Mortality, Outcome

## Abstract

**Background:**

There have been several meta-analyses evaluating the effect of n-3 polyunsaturated fatty acids (PUFAs) in critically ill patients, but of these, none focused on patients with systemic inflammatory response syndrome (SIRS). The objective of this meta-analysis was to evaluate the effect of omega-3 fatty acids (n-3 FAs) on this narrow subset.

**Methods:**

All relevant articles were searched on MEDLINE, EMBASE, SpringerLink, and the Cochrane Database of Systematic Reviews from 1990 to 2014. Meta-analyses were used to evaluate risk ratios and mean differences with 95% confidence intervals between the n-3 PUFA group and the control group. Subgroup analyses were conducted in terms of the route of fish oil.

**Results:**

Nine randomized controlled trials (RCTs) with 783 adult patients were included in this study. Compared with control groups, n-3 FA provision can significantly reduce the incidence of mortality (RR: 0.77 [0.60, 0.97]; P = 0.03; I^2^ = 0%). Secondary outcomes showed no significant differences between groups except for shorter length of hospital stay (weighted mean difference: −10.56 [−19.76, −1.36], p < 0.00001, I^2^ = 99%).

**Conclusions:**

Overall, this meta-analysis from RCTs indicates that provision of n-3 PUFAs has a therapeutic effect on survival rate in patients with SIRS.

## Introduction

Systemic inflammatory response syndrome (SIRS) is an inflammatory state affecting the whole body. Frequently, it is the result of the body’s immune response to an infectious or noninfectious insult [1-3]. It is related to sepsis, a condition in which individuals meet criteria and have a known infection. SIRS is characterized by systemic inflammation, organ dysfunction, and organ failure such as acute lung injury, acute kidney injury, shock, and multiple organ dysfunction syndrome. Although antibiotics and various supportive therapies are constantly being developed, it remains one of the major causes of death in critically ill patients [3]. Thus, learning how to effectively control the body’s systemic inflammation is an important research target for the treatment of SIRS.

Omega-3 polyunsaturated fatty acids (n-3 PUFAs) are essential fatty acids and are considered as providing potential health benefits [4,5]. Supplementation with n-3 PUFAs, either parenterally or enterally, is thought to be potentially beneficial in modulating inflammatory processes [6]. In 2009, the European Society for Parenteral and Enteral Nutrition (ESPEN) guidelines recommended the use of fish oil in critical patients, especially in those with acute lung injury, sepsis, or SIRS [7]. However, in the OMEGA trial, the therapeutic effect on acute lung injury disappeared [8], making the use of n-3 fish oil a topic of controversy [9].

Recently, many randomized controlled trials (RCTs) have investigated the effect and safety of fish oil on SIRS or sepsis [10-18]. However, interpretation of these results is problematic owing to the small sample sizes and methodic limitations. Moreover, several recent RCTs reported conflicting data. As a result of the advantages and disadvantages of n-3 PUFAs, we performed a systematic review of RCTs with meta-analysis to investigate the effect of n-3 PUFA supplementation in adult patients with SIRS or sepsis.

## Results

### Study selection and risk of bias

**Figure 1:**
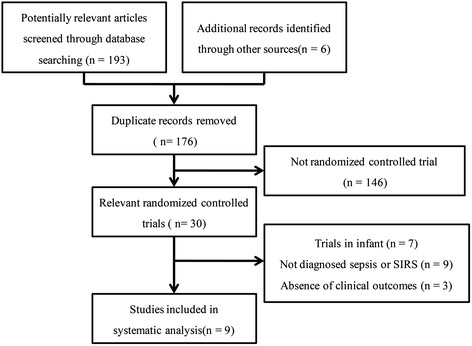
Flowchart of trial selection process. SIRS – systemic inflammatory response syndrome.

A total of 192 potentially relevant studies were retrieved, and six additional records were identified through other sources. The process of selecting relevant trials is described in Figure 1. Finally, nine citations of RCTs with a total of 783 patients were included in our meta-analysis [10-18]. Among these trials, two were conducted in Spain, two in Brazil, and one each in Portugal, Germany, United States, Egypt, and Taiwan. In four trials, fish oil was administered intravenously, while in the others, it was administered enterally. The characteristics of the included trials are shown in Table 1.

**Table 1:** **Characteristics of included trials**

**Author/year**	**Blind**	**Intervention**	**n-3/n-6 ratio**	**Dose and form of fish oil**	**Route**	**No. of patients (valued/enrolled)**	**Days**	**Severity of illness**	**Mortality**
Barbosa 2011 [10]	Single-blind	FO + LCT + MCT vs. LCT + MCT	FO group 1:4	Dose: not mentioned	Parenteral	23/25	5	SAPS II: 47.5 ± 5 vs. 41.6 ± 6.5	28-day mortality: 4/13 vs. 4/10
			Control group (no n-3 FO)						
				Form: 2.5% EPA 1.1% DHA					
Cristóbal 2000 [11]	Single-blind	Impact. vs. control	Not reported	Not mentioned	Enteral	176/181	≥4	APACHE II: 18.4 ± 5.6 vs. 17.9 ± 5.2	Hospital mortality: 17/89 vs. 28/87
Friesecke 2008 [12]	Double-blind	FO + LCT + MCT vs. LCT + MCT	FO group 1:2	Not mentioned	Parenteral	80/114	7	SAPS II: 49 ± 18 vs. 54 ± 17	28-day mortality: 10/42 vs. 8/38
			Control group 1:7						
Grau-Carmona 2011 [13]	Single-blind	EPA + GLA vs. control	FO group 1: 1.5	Dose: not mentioned	Enteral	132/160	7–22	SOFA: 9 (7–11) vs. 9 (8–11) ^*a*^ APACHE II: 19 (16–24) vs. 19 (16–23) ^*a*^	28-day mortality: 11/61 vs. 11/71
			Control group 1: 5.8						
				Form: 2.5% EPA 0.08%GLA					
Hall 2014 [14]	No-blind	FO + stand care vs. stand care	Not mentioned (Omegaven was used singly in FO group)	Dose: 0.2 g/kg/d	Parenteral	60/87	≤14	APACHE II: 19.1 ± 6.7 vs. 17.9 ± 6.2 SOFA: 7.2 ± 3.0 vs. 7.6 ± 3.2	Hospital mortality: 4/30 vs. 9/34
				Form: 1.25%–2.82% EPA 1.44%–3.09% GLA					
Hosny 2013 [15]	Not mention	high dose FO vs. low dose FO vs. control	high FO group 1:6	Dose: high FA 9 g/d low FA 3 g/d control 0 Form: not mentioned	Enteral	75/not reported	7	SOFA: 3.7 ± 0.96 vs. 3.3 ± 1.31 vs. 3.6 ± 0.87	28-day mortality: 8/25 vs.11/25 vs. 10/25
			low FO group 1:20						
			control group (no n-3 FO)						
Khor 2011 [16]	Double-blind	FO vs. placebo	Not mentioned (Omegaven® was used singly in FO group)	Dose: 10 g/d	Parenteral	27/28	5	APACHE II: 19.3 ± 7.8 vs. 16.3 ± 7.2 SAPS II: 52.9 ± 22.5 vs. 48.4 ± 15.1	28-day mortality: 0/14 vs. 0/13
				Form: 1.25%–2.82% EPA 1.44%–3.09% GLA					
Pontes-Arruda 2006 [17]	Double-blind	EPA + GLA vs. control	FO group 1:1.85	Dose: not mentioned	Enteral	103/165	4-7	SOFA: 8.8 ± 0.9 vs. 8.6 ± 0.8	28-day mortality: 18/55 vs. 25/48
			control group 1:3.8						
				Form: 4.5% EPA 4.3% GLA 2% DHA					
Pontes-Arruda 2011 [18]	Double-blind	EPA + GLA vs. control	FO group 1:1.85	Dose: not mentioned	Enteral	53 vs. 53	7	APACHE II: 19.5 (17–25) vs. 20 (16–23) ^*a*^ SOFA: 5.5 (4–9) vs. 6 (4–8) ^*a*^	Not reported
			control group 1:3.8						
				Form: 4.5% EPA 4.3% GLA 2% DHA					

Values are presented as mean ± standard deviation unless indicated otherwise.

*a* – median (interquartile range), APACHE – acute physiology and chronic health evaluation, DHA – docosahexaenoic acid, EPA – eicosapentaenoic acid, FO – fish oil, GLA – gamma-linolenic acid, LCT – long chain triglycerides.

MCT – medium chain triglycerides, SAPS – simplified acute physiology score, SOFA – Sequential organ failure assessment.

**Figure 2:**
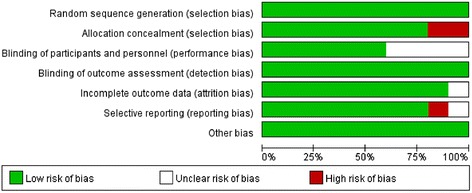
Risk of bias graph: Review of authors’ judgments about each risk of bias item, presented as percentages across all included studies.

**Figure 3:**
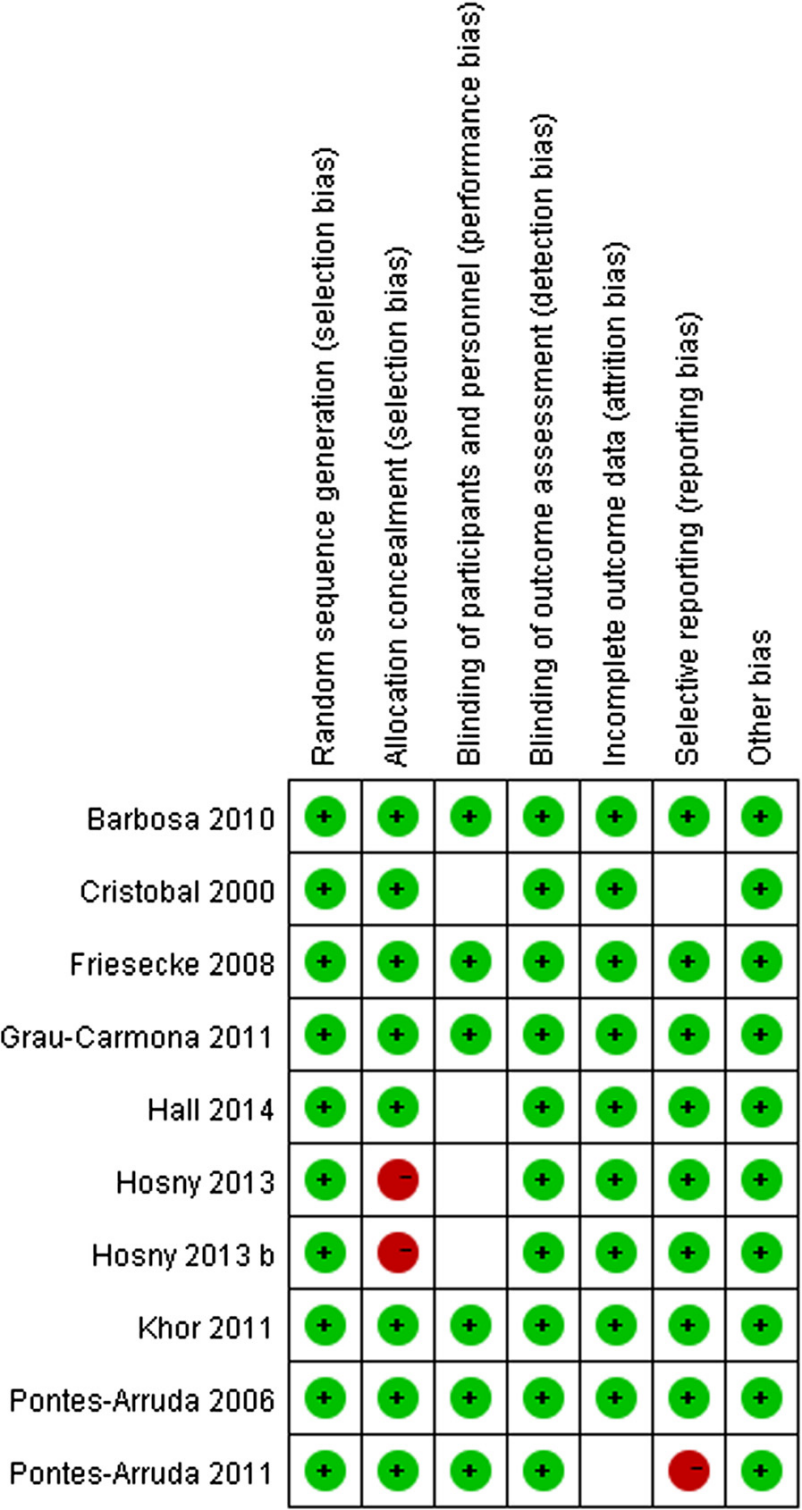
Risk of bias summary: Review of authors’ judgments about each risk of bias item for each included study.

Among the selected trials, four were double blind, three were single blind, and two were open-label trials. All the risks of bias item for each included study were presented in Figures 2 and 3. Randomized sequence and allocation sequence concealment were conducted adequately, except for the 2013 trial [16]. One trial was evaluated as having a high risk of reporting bias because of the lack of morbidity [17].

### Clinical outcomes

**Figure 4:**
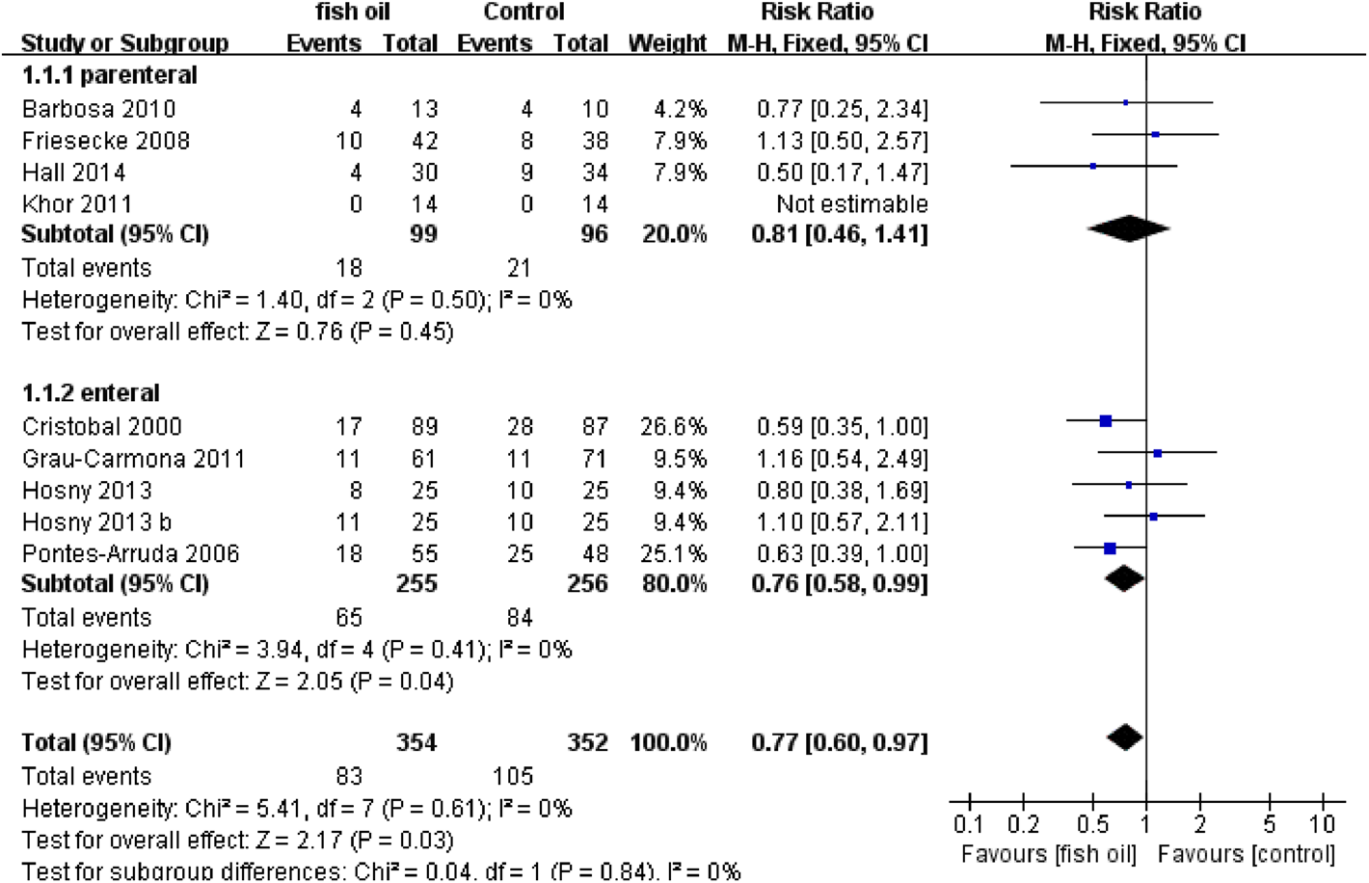
Forest plot of comparison: Effect of n-3 fish oil supplementation on mortality. CI – confidence interval, RR – relative risk.

**Figure 5:**
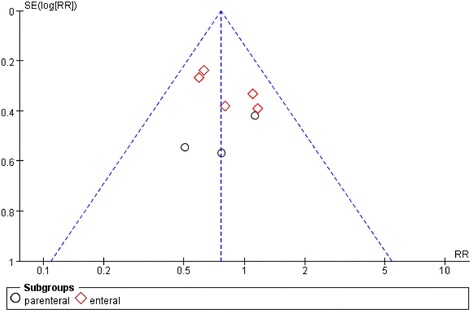
Funnel plot of comparison: Effect of n-3 fish oil supplementation on mortality. RR – relative risk.

The results of the meta-analysis are demonstrated in Table 2. The primary meta-analysis outcome of eight trials with 706 participants was that the use of n-3 PUFAs significantly reduced mortality in patients with SIRS, as shown in Figure 4 (RR: 0.77 [0.60, 0.97]; P = 0.03; I^2^ = 0%). The funnel plot is presented in Figure 5.

**Table 2:** **Meta-analysis of included trials**

**Outcome of interest**	**No. of trials**	**No. of patients**	**Fixed effect model**			**Random effects model**		
			**RR/WMD (95% CI)**	**P**	**I** ^**2**^	**RR/WMD (95% CI)**	**P**	**I** ^**2**^
Mortality	8	706	0.77 [0.60, 0.97]	0.03	0%	-	-	-
Days on ventilation	6	567	−3.57 [−4.22, −2.91]	<0.0001	95%	−2.61 [6.29,1.07]	0.17	95%
Ventilator-free days	3	243	7.29 [6.88, 7.71]	<0.0001	96%	3.09 [−3.34, 9.51]	0.35	96%
LOS-ICU	8	656	−2.76 [−3.42, −2.10]	<0.0001	92%	−1.35 [−4.12, 1.43]	0.34	92%
LOS-H	4	217	−5.04 [−5.86, −4.22]	<0.0001	99%	−10.56 [−19.76, −1.36]	0.02	99%

LOS-H – length of stay in hospital, LOS-ICU – length of stay in intensive care unit, RR – relative risk, WMD – weighted mean difference.

**Figure 6:**
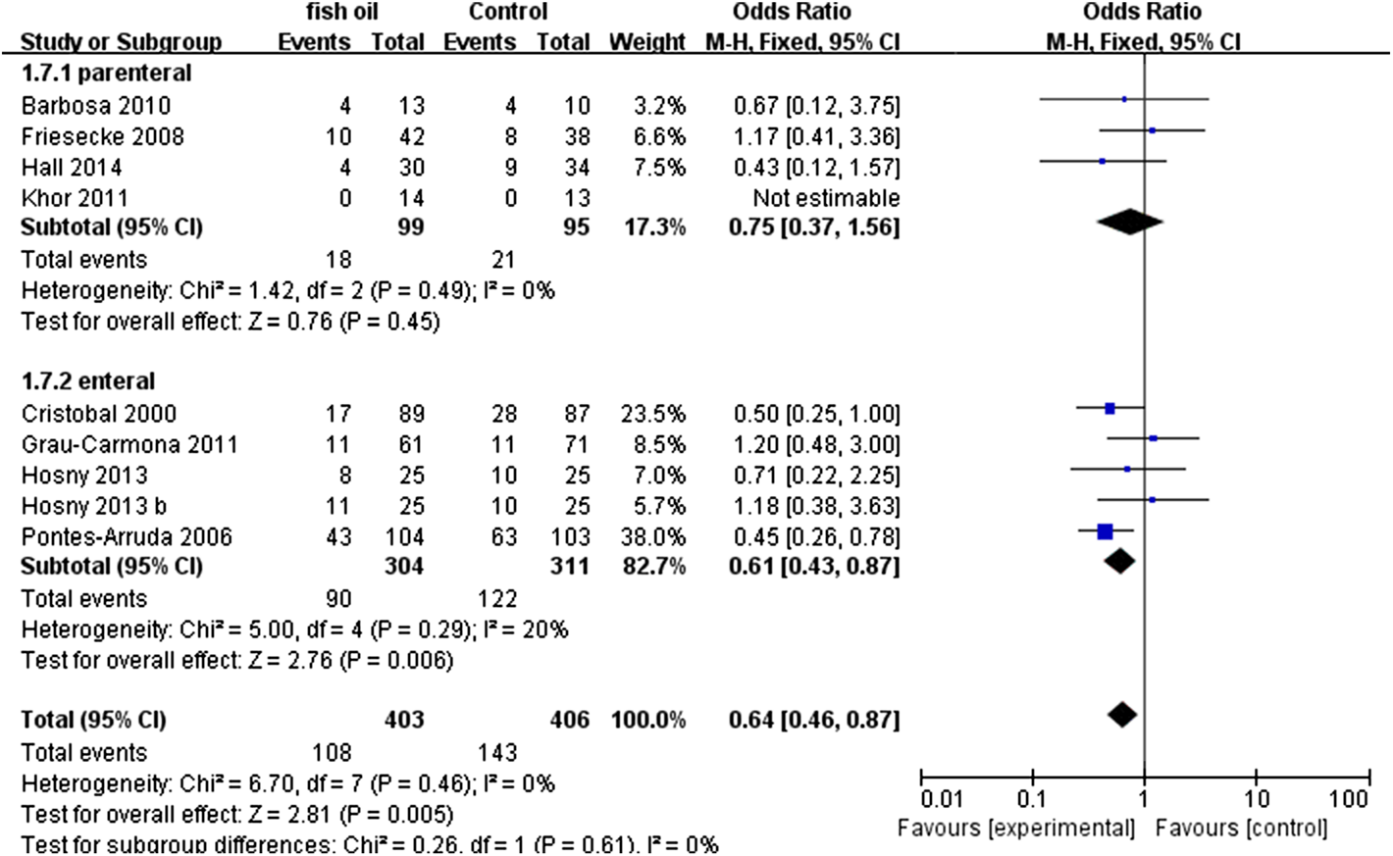
Forest plot of comparison: Effect of n-3 fish oil supplementation on assumed total mortality. CI – confidence interval, RR – relative risk.

Besides, with the limitation in Pontes-Arruda, *et al.* [17] that several deceased patients were assigned into the “withdrew” group, we reanalyzed in terms of an assumption that the patients who were still alive when they were withdrawn from the study have the same mortality as the patients continuing in Pontes-Arruda, *et al.* [17]. The death rates were 63/103 in the control group and 43/104 in the fish oil group, respectively. The result was the same as before: n-3 PUFAs significantly reduced mortality in patients with SIRS (RR: 0.64 [0.46, 0.87]; P = 0.005; I^2^ = 0%) (Figure 6).

**Figure 7:**
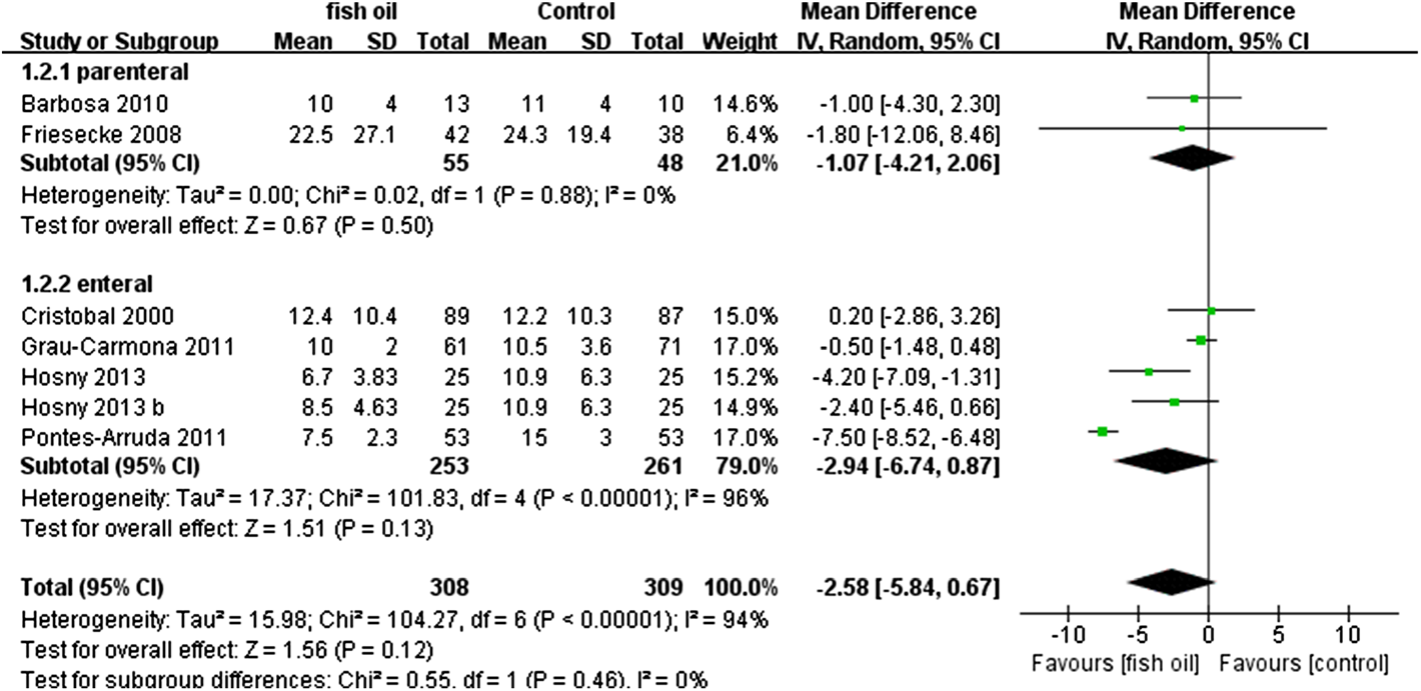
Forest plot of comparison: Effect of n-3 fish oil supplementation on days on ventilation. CI – confidence interval, MD – mean difference, SD – standard deviation.

**Figure 8:**
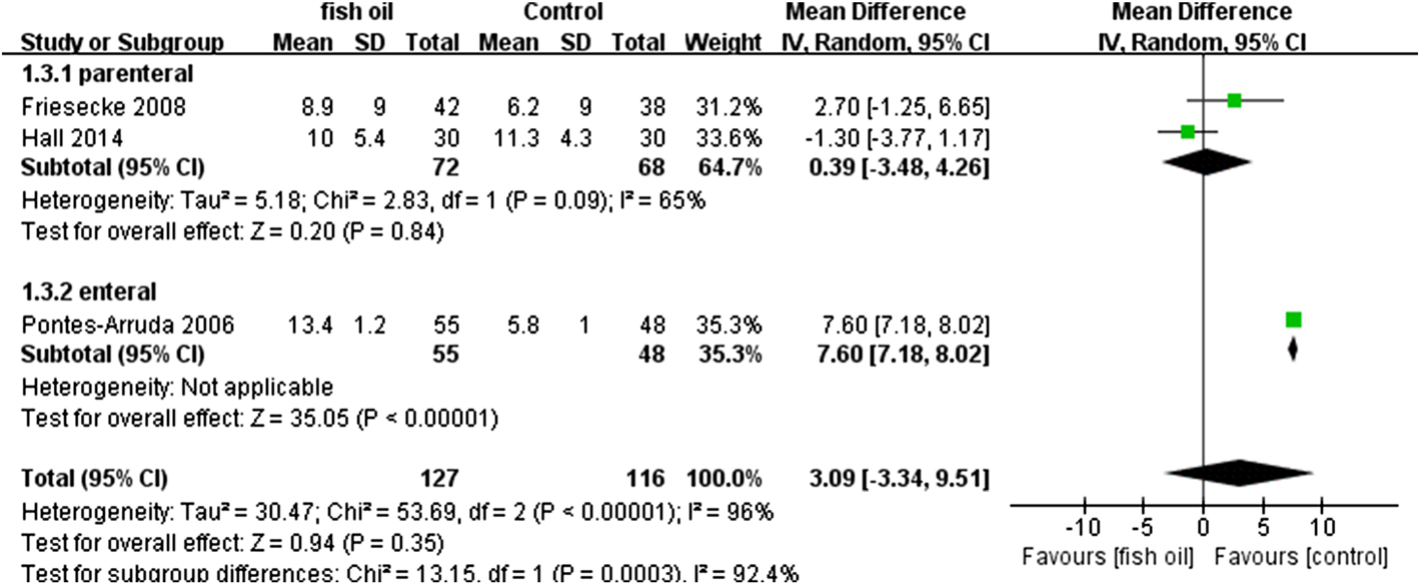
Forest plot of comparison: Effect of n-3 fish oil supplementation on ventilator-free days. CI – confidence interval, MD – mean difference, SD – standard deviation.

**Figure 9:**
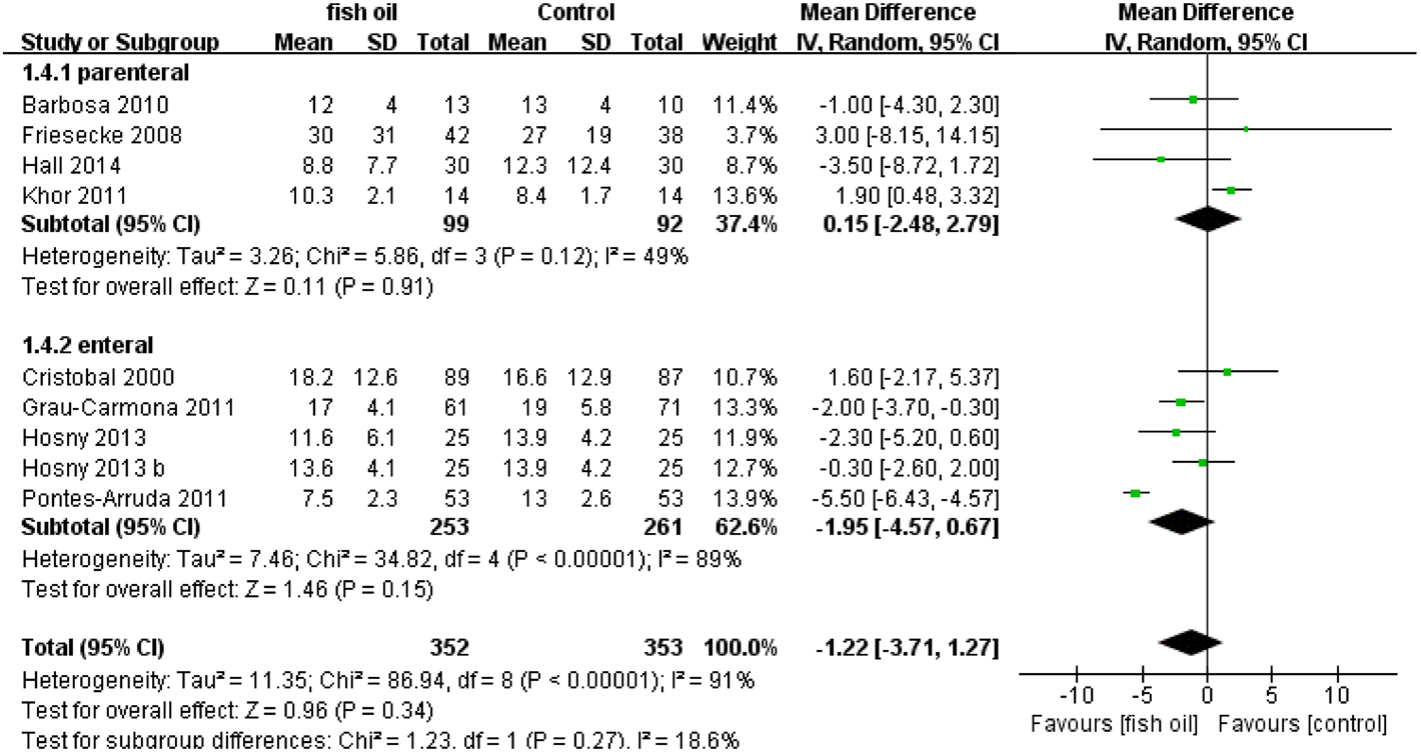
Forest plot of comparison: Effect of n-3 fish oil supplementation on LOS-ICU. CI – confidence interval, LOS-ICU – length of stay in intensive care unit, MD – mean difference, SD – standard deviation.

**Figure 10:**
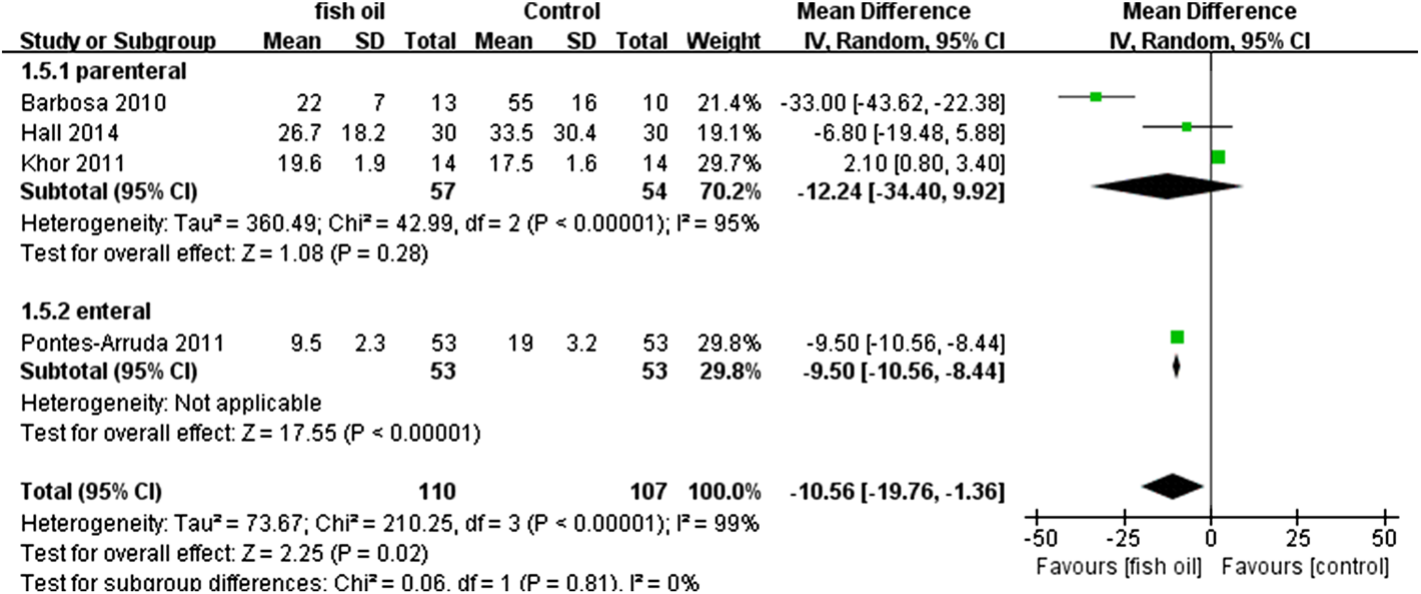
Forest plot of comparison: Effect of n-3 fish oil supplementation on LOS-H. CI – confidence interval, LOS-H – length of stay in hospital, MD – mean difference, SD – standard deviation.

When we evaluated secondary outcomes including days on ventilation, ventilator-free days, LOS-ICU, and LOS-H, the heterogeneity was significant. The n-3 FA had therapeutic effects in fixed models: days on ventilation (WMD: −3.57 [−4.22, −2.91], P < 0.00001, I^2^ = 95%), ventilator-free days (WMD: 7.29 [6.88, 7.71], p < 0.00001, I^2^ = 96%), LOS-ICU (WMD: −2.76 [−3.42, −2.10], P < 0.00001, I^2^ = 92%), and LOS-H (WMD: −5.04 [−5.86, −4.22], P < 0.00001, I^2^ = 99%). However, these effects were no longer present in a random effects model except for shortening the LOS-H: days on ventilation (WMD: −2.61 [6.29,1.07], P = 0.17, I^2^ = 95%), ventilator-free days (WMD: 3.09 [−3.34,9.51], P = 0.35, I^2^ = 96%), LOS-ICU (WMD: −1.35 [−4.12, 1.43], P = 0.34, I^2^ = 92%), and LOS-H (WMD: −10.56 [−19.76, −1.36], P < 0.02, I^2^ = 99%). All the results in random effects models are presented in Figures 7, 8, 9 and 10.

### Subgroup analysis

A subgroup analysis was performed to compare the difference between parenteral and enteral routes, but no difference was observed for mortality (P = 0.84, I^2^ = 0%), days on ventilation (P = 0.46, I^2^ = 0%), LOS-ICU (P = 0.27, I^2^ = 18.6%) and LOS-H (P = 0.81, I^2^ = 0%). By contrast, the difference for ventilator-free days was significant (P = 0.0003, I^2^ = 92.4%), which was likely to be caused by the small sample size (n = 4).

## Discussion

According to the RCT meta-analysis, n-3 PUFA supplementation has a therapeutic effect on patients with sepsis or SIRS because it can effectively reduce mortality without heterogeneity. It also can shorten the LOS-H. However, the n-3 FA group does not statistically differ from placebo or control subjects in terms of days on ventilation, ventilator-free days, and LOS-ICU. Significant heterogeneity among these trials precluded the pooling of results.

Although the anti-inflammatory function of n-3 PUFAs is not known to be associated with the route of supplementation, we performed a subgroup analysis. This result is consistent with the consensus in terms of mortality, days on ventilation, ventilator-free days, LOS-ICU, and LOS-H. The only difference that is not as reliable is that of ventilator-free days, because of its small sample size and contradiction with days on ventilation.

It is crucial that, in the provision of nutrition support, strategies should be adopted to optimize benefit and minimize potential harm. Recently, immunonutrition has been extensively applied in clinical practice, especially with respect to n-3 FAs in critically ill patients. Fish oil intake, which provides eicosapentaenoic acid (EPA) and docosahexaenoic acid (DHA), results in a higher proportion of EPA and DHA and a lower proportion of arachidonic acid in the cell membrane, thereby decreasing the synthesis of inflammatory cytokines such as tumor necrosis factor-alpha, interleukin-6, and interleukin-8 [19,20]. Early studies confirmed that fish oil can reduce the mortality of critically ill patients with acute lung injury (ALI), sepsis, and SIRS [12.18]. A decrease in length of stay without any other detectable effects has also been shown in surgical patients requiring intensive care after five days of n-3 FA emulsion [21]. Fish oil use in patients with severe pancreatitis also resulted in decreased inflammatory response, as well as improved respiratory function [22,23]. Friesecke *et al.* revealed an opposing opinion, reporting that use of a mixed long-chain triglyceride (LCT)/medium-chain triglyceride (MCT)/fish oil lipid emulsion in critically ill ICU patients had no improvement on clinical outcomes including infections, ventilation days, LOS-ICU, or LOS-H compared with MCT/LCT. In fact, the ESPEN guidelines show a Grade B recommendation for the infusion of fish oil in critically ill patients [7,13].

In recent years, some studies have yielded different results, especially the OMEGA study. This study for fish oil in the treatment of patients with ALI has subversive significance. The two articles about n-3 PUFAs in patients with ALI/acute respiratory distress syndrome (ARDS) also point out that if the OMEGA study were included, the heterogeneity of the research would be obvious (I^2^ = 31%), and the beneficial effect of fish oil for the treatment of patients would have no clinical significance (P = 0.36) [9]. If the OMEGA study were excluded, however, the heterogeneity would disappear (I^2^ = 0%), and we would be able to positively confirm that fish oil does reduce mortality, accompanied by a decline in reliability [24]. The two authors made different choices, which resulted in two opposite sets of conclusions and recommendations.

In addition, the use of fish oil may affect clinical outcomes, although the traditional view has been that oral and intravenous fish oil only differ in effect speed and utilization degree. A meta-analysis in 2014 confirmed that parenteral fish oil had a tendency to reduce the mortality of ICU patients without heterogeneity (P = 0.08) [25]. After we compared the result with the investigation conclusions for ALI/ARDS, concerns were raised as to whether the use of methods (enteral vs. parenteral) and disease type (ALI/ARDS vs. sepsis/SIRS) had a large influence on response to treatment.

Our meta-analysis answered this question. It indicated that mortality was significantly reduced by the n-3 FA supplementation, which was in line with the result of Marik *et al.* [26]. There are, however, several possible explanations for the former question. First, n-3 FA can effectively suppress systemic inflammation. Second, there is no significant difference between the parenteral and enteral groups, which proves that the method of use does not influence the anti-inflammatory effects of fish oil. In addition, the parenteral fish oil in the total group cannot obviously improve mortality, and this significant result is probably a false negative result because the number of people enrolled is small (n = 195) [27]. We need more clinical research detailing parenteral n-3 FA in patients with sepsis or SIRS to confirm this conclusion.

However, some limitations of this meta-analysis should be considered. First, all the trials included in this study are small sample clinical trials, and the biggest single research study enrolled only 176 participators. Even though the funnel plot figure of mortality confirmed equal research distribution without obvious bias, the credibility of the results still needs to be considered with caution, and we need more large sample clinical studies to solve this problem. Second, the other results have high heterogeneity. After using subgroup analysis in terms of intervention or illness severity, we failed to find the source of heterogeneity. It’s likely that the included trials differed in nutrition formula, administration, and treatment protocol. Moreover, the primary cause of SIRS is various, and most of trials reported multiple causes. Thus, caution should be taken when interpreting the results, and.high heterogeneity greatly reduces the credibility of these conclusions.

## Conclusions

In conclusion, when nutrition is prepared for patients with sepsis or SIRS, supplemental n-3 PUFAs is associated with lower mortality, and, if possible, should be considered the standard choice for nutritional support. It also has the potential ability to shorten the LOS-H. Future large-scale, high-quality RCTs are still required to clarify the effectiveness of n-3 FA supplementation in sepsis and SIRS, especially by the intravenous route.

## Methods

### Study Identification

Two researchers independently searched the published literature without language restrictions using the keywords “omega-3 fatty acids,” “fish oils,” “sepsis,” “SIRS,” “critical illness,” and their analogues on MEDLINE, EMBASE, SpringerLink, and the Cochrane Database of Systematic Reviews from 1990 to 2014. Potentially relevant studies from the identified reports were searched to find relevant trials. Abstracts from scientific meetings were accepted in the review if the data were available to complete the analysis.

### Inclusion and Exclusion Criteria

Original studies were selected for inclusion in the review process only if they met the following conditions: (1) the study design was consistent with clinical RCTs; (2) the study population comprised patients > 14 years of age who were diagnosed with sepsis or SIRS; (3) the intervention was fish oil (enteral, parenteral, or both) versus control (other lipid or placebo); and (4) clinical outcomes were prespecified, including one of the following: mortality, days on ventilation, ventilator-free days, length of stay in intensive care unit (LOS-ICU), and in hospital (LOS-H). Studies that evaluated only biochemical, metabolic, immunologic, or nutritional outcomes were not included. Furthermore, if n-3 PUFAs were administered for less than 24 hours, the article was to be excluded.

### Data extraction

All original studies were retrieved in duplicate by two independent reviewers (Wan Xiao and Gao Xuejin), using a data abstraction form. The following data were extracted from each study: authors, year of publication, study design, patient characteristics, study methodology (e.g., inclusion/exclusion criteria, nutrition administration protocol, randomization, and blinding), intervention (e.g., duration, form, and daily dose), and outcome measures. We attempted to contact the authors of included studies and requested additional information not contained in published articles.

### Assessment of risk bias

All original data were evaluated independently by two reviewers. The risk bias of included trials was assessed using the components recommended by the Cochrane Collaboration: random sequence generation; allocation concealment; blinding of participants, personnel, and outcome assessors; incomplete outcome data; selective reporting; and other sources of bias [28]. Disagreement on the individual risk bias of each of the categories was resolved by consensus between both reviewers.

### Statistical analysis

The analyses were performed in RevMan 5.2 (The Nordic Cochrane Centre, Copenhagen, Denmark). As part of the results were abnormally distributed, we represented the data as mean ± SD [29]. If hospital mortality was not reported, we used 28-day or 60-day mortality instead. The differences between the fish oil group and control group were expressed by the relative risk (RR) and the weighted mean difference (WMD) with 95% confidence intervals (CI) for dichotomous and continuous outcomes, respectively. The presence of heterogeneity was quantified using the I^2^ statistic. If without statistical heterogeneity (I^2^ = 0%), a fixed-effect model was used; otherwise, both fixed-effect model and random effects models were used [30]. Graphic exploration with a funnel plot was used to visually evaluate the small trial bias.

## Abbreviations

CI: Confidence interval
n-3 PUFAs: Omega-3 polyunsaturated fatty acids
SIRS: Systemic inflammatory response syndrome
ESPEN: the European Society for Parenteral and Enteral Nutrition
RCT: Randomized controlled trials
LOS-ICU: Length of stay in intensive care unit
LOS-H: length of stay in hospital
RR: Relative risk
WMD: Weighted mean difference
EPA: Eicosapentaenoic acid
DHA: Docosahexaenoic acid
LCT: Long-chain triglyceride
MCT: Medium-chain triglyceride
ARDS: Acute respiratory distress syndrome
